# The efficacy of attendance and semi-attendance group cognitive-behavioral therapy (CBT) on the anxiety disorders of adolescent girls

**Published:** 2010

**Authors:** Afsaneh Karbasi, Soroor Arman, Mohamad Reza Maracy

**Affiliations:** a Child Psychiatrist, Behavioral Sciences Research Center, Isfahan University of Medical Sciences, Isfahan, Iran; b Associate Professor, Behavioral Sciences Research Center, Isfahan University of Medical Sciences, Isfahan, Iran

**Keywords:** Anxiety Disorder, Therapy, Cognitive Beavior, Group Therapy

## Abstract

**BACKGROUND:**

Anxiety disorders are one of the most psychiatric disorders in children and adolescents that can cause long life functional disability. The first line treatment for this disorder is cognitive behavioral therapy that has primary, secondary and tertiary preventive effect, but is expensive and long time. Today there is some effort to find short term, group, semi-attendance and low cost therapies.

**METHODS:**

Subjects were 42 girls (12- 17 y) with at least one anxiety disorder according to DSM-IV-TR with their parents who were divided into two groups randomly: group A which participated in 8 sessions and group B which participated in 4 sessions and the contents of sessions 3, 4, 6, and 7 were recorded on a CD for them. The tests used in this study were: SCARED, CATS, CAIS-C, CAIS-P, conducted before (T0), just after (T1) and three months after the treatment (T2). The collected data were analyzed by multivariate analysis of covariance test using SPSS software package, version 15.0.

**RESULTS:**

There was no significant difference between efficacy of semi-attendance group CBT and attendance group CBT in T0, T1 and T2 according to 4 tests (p = 0.311). The difference between the scores of these tests between T0 andT1 and T0 and T2 was significant in both groups (p < 0.001) but the difference between T1 and T2 was not significant. (p = 0.771).

**CONCLUSIONS:**

The efficacy of semi-attendance group CBT and attendance group CBT is similar and would sustain after 3 months.

Anxiety disorders are among common psychiatric disorders of children and adolescents.^1^ Epidemiologic studies have reported the incidence of anxiety disorder to be 5.7-17.7% at this age.^2^–^4^ Childhood anxiety disorders are severely disabling, inducing functional disability at home, school and in relation with peers and reduction of self confidence.^3^–^5^ On the other hand, this disorder can influence the function in adulthood mostly causing depression, drug abuse, suicide, social withdrawal and a need for hospitalization.^6^ There is also a high correlation between anxiety and externalizing disorders.^7^ Longitudinal studies have shown that childhood anxiety disorders are resistant and chronic.^6^,^7^ It gets even more prevalent among girls in adulthood,^8^ causing a lot of trouble for health care systems.^3^ The treatment of these disorders has been effective by Selective Serotonin Reuptake Inhibitors (SSRIs) but with unknown long term side effects among growing children.^9^ Since this medication can result in hyperactivity, GI problem or amotivational syndrome, most of the parents reject that.^3^ On the other hand, in spite of the fact that this medication results in no change in thought and behavior, adolescents usually avoid taking the medication and prefer to solve their problem with non medication treatments.^10^

The first article on the efficacy of cognitive behavioral therapy on anxiety of children and adolescents were written over ten years ago.^6^ Cognitive Behavioral Therapy (CBT) has been proved to be the first line treatment in various meta-analyses,^11^,^12^ and it has been effective even at preschool age.^12^ Individual, group, parental/familial and school based cognitive behavioral therapies have shown equal results^3^–^5^,^11^,^12^ (effect size = 0.58-0.94, 56-65%).^2^,^6^,^9^,^13^ Permanency of individual and group treatments have been proved to be 6-8 years and one year respectively.^12^–^14^

The combination of this treatment with medication in acute conditions such as school refusal, social isolation and low response to psychotherapy has been reported effective.^12^ Early diagnosis and intervention of anxiety disorders can prevent negative outcomes of non treated anxiety.^5^,^11^ This treatment has been proved to play a role in primary prevention like low incidence, in secondary prevention like disappearing all signs and in tertiary prevention like decrease of severity and length of signs.^6^ The preventive role of this treatment has been studied up to two years in some researches.^6^

Since these disorders are more prevalent among low socioeconomic classes of society,^1^ and as parental/familial and individual cognitive behavioral therapy costs a lot, the trend of studies has been driven to lower cost therapies such as school based or group cognitive behavioral therapy.^15^

Nowadays, there are studies being carried out on distance methods through computer or internet programs with optimistic results, although some experts believe that face to face communication is necessary for CBT.^15^,^16^

This study has been designed and conducted to find an effective, harmless, low cost, short term and attractive treatment for adolescents through which the patient plays an active role in controlling his/her signs.

## Methods

This is a randomized clinical trial that was specially conducted for one year from September 2008 to November 2009. The study was carried out on child/adolescent sub-specialty psychiatry clinic in Noor Hospital of Isfahan University of Medical Sciences, Iran. First, forty four adolescent girls aged from 12 to 17 years, who referred to child/adolescent clinic, diagnosed with at least one anxiety disorder based on DSM-IV-TR (Diagnostic & Statistical Manual of Mental Disorders IV-Text Revised) by a sub-specialist of pediatric psychiatry, were selected. Then, semi structured interview of ADIS (ADIS^16^ : Anxiety Disorders Interview Schedule for DSM-IV, Child Version) was separately conducted for the adolescents and their parents. The participants with IQ lower than 80, learning disorder, drug abuse, psychosis, serious mental or physical problems and low interest in continuing the study were excluded from this study. Taking medication with no change in dosage in the past one month was ignored.^17^ Written consent based on Helsinki ethical protocol was taken from one of the samples’ parents. All participants, based on their referral to clinic, were randomly put in either attendance intervention (group A) or semi-attendance (group B). Pre treatment measurements regarding the signs of anxiety, automatic anxiety thoughts, and anxiety function interference from adolescents’ and parents’ viewpoint were separately performed by following questionnaires:

1. SCARED-R (SCARED-R: Screen for Child Anxiety Related Emotional Disorders, Revised): That is the best self multi dimensional measurement questionnaire with 66 items for children and adolescents’ anxiety disorders^18^,^19^ with reliability of 0.70-0.90 and high convergent divergent validity^20^–^22^ that is an appropriate sensitive psychometric tool to treatment effects.
2. CATS (CATS: Children’s Automatic Thoughts Scale): That is a self-report tool for ages 8-17 years with 40 items to measure automatic thoughts within recent week. Its internal consistency, including total score and score of its four subscales is so high. Its test-retest reliability is acceptable up to 1-3 months and its discriminative validity between depression, anxiety and behavioral disorders is acceptable.^23^
3. CAIS-C (CAIS-C: Children’s Anxiety Interference Scale (Child form)): That is a self-report scale to investigate the effect of fears and worries on different domains of life.
4. CAIS-P (CAIS-P: Children’s Anxiety Interference Scale (Parent form)): That measures anxiety function interference of children in different domains of their parents’ life.^1^

The size of each group was 10 subjects. For the attendance group, there were 8 sessions held weekly.

The protocol used in these sessions was taken from BRAVE program of Australian Macquarie University Anxiety Research Unit that is a group cognitive behavioral therapy for adolescents aged 13-17 years with various anxiety disorders.^16^ The content of sessions (psycho education, relaxation, relation of thought-emotion-action, substitution of unhelpful thoughts with helpful ones, graded exposure, problem solving, and self reward) were prepared and modified and given to adolescents and their parents in form of separate illustrated booklets. In the end of the sessions, there was homework for adolescents and parents to take home.

In the semi-attendance group, there were 4 sessions and the content of other four sessions was given to the patients in form of CDs. The CDs included some films which were taken from sessions 3, 4, 6, and 7 in addition to some diagrams and photos. The CD was developed by the researches and it took one hour for each session.

The subjects were followed up through phone calls. The sessions were held for 75 minutes. Adolescents and their parents attended a shared session for 15 minutes, then they had parallel and separate sessions, each for 45 minutes with a therapist and in the last 15 minutes again they were in a session together. The sessions of adolescents and parents were held simultaneously in order to facilitate families’ attendance in treatment and play therapy room was ready to those with small children. The therapists performance were quite identical as they were recorded and re-checked by the researchers. All tests were taken before treatment (T_0_), just after finishing the program (T_1_) and three months after the treatment (T _2_). The obtained data were analyzed by multivariate analysis of covariance test using SPSS software package, version 15.0.

## Results

There were 42 adolescent girls with anxiety aged from 12-17 years attending this study. There were two missing subjects in each group due to low interest to join the research. The mean age in group A and group B were 14.2 ± 1.6 and 15.1 ± 1.5 years, respectively with no significant difference (p > 0.05). Regarding the frequency distribution of various psychiatric disorders, there were significant difference only for panic disorder (p = 0.011) and eating disorder (p = 0.011). The mean ± standard deviation (SD) of the scores in SCARED, CATS, CAIS-C and CAIS-P questionnaire before, just after finishing and three months after treatment in both groups have been presented in table 1.

**Table 1:** Mean (SD) of the anxiety disorder scores using the four questionnaire in the attendance (A) and semi attendance (B) groups in three different times

		A		B	
		
		n	Mean (SD)	n	Mean (SD)
SCARED-R questionnaire	Before	22	106.2 (36.3)	22	109.3 (37.8)
	(T_0_)				
	After	20	81.4 (21.4)	20	77.8 (21.9)
	(T_1_)				
	After 3 months	20	75.8 (28.9)	20	69.9 (22.5)
	(T_2_)				
CATS questionnaire	Before	22	56 (28.4)	22	51.9 (28.1)
	(T_0_)				
	After	20	43.8 (14.1)	20	38.1 (10.7)
	(T_1_)				
	After 3 months	20	36.9 (15.3)	20	38 (11.3)
	(T_2_)				
CATS-C questionnaire	Before	20	20.2 (8.5)	20	18.9 (9.3)
	(T_0_)				
	After	20	14.6 (7.4)	20	11.5 (4.5)
	(T_1_)				
	After 3 months	20	14.7 (7.8)	20	11.3 (6.6)
	(T_2_)				
CAIS-P questionnaire	Before	20	31.1 (12.1)	20	23.4 (10.3)	
	(T_0_)				
	After	20	23 (10.8)	20	16.5 (7.3)
	(T_1_)				
	After 3 months	20	21.8 (10)	20	18.2 (6)
	(T_2_)				

**Figure 1:**
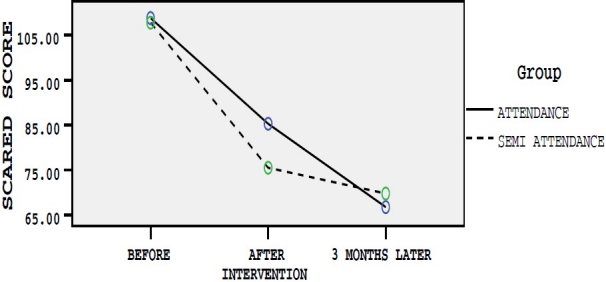
Mean scores of SCARED questionnaire in three different times in group A and B

**Figure 2:**
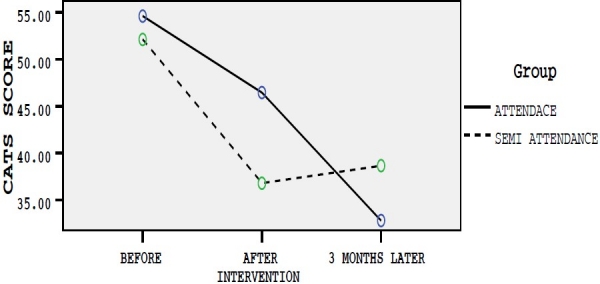
Mean scores of CATS questionnaire in three different times in group A and B

**Figure 3:**
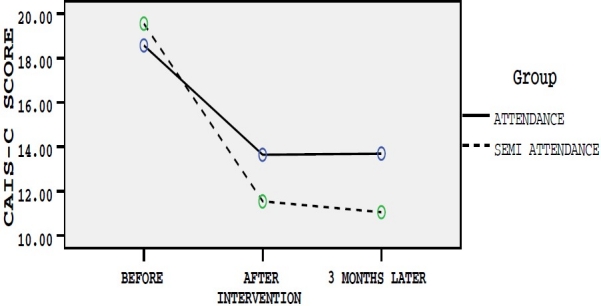
Mean scores of CAIS-C questionnaire in three different times in group A and B

**Figure 4:**
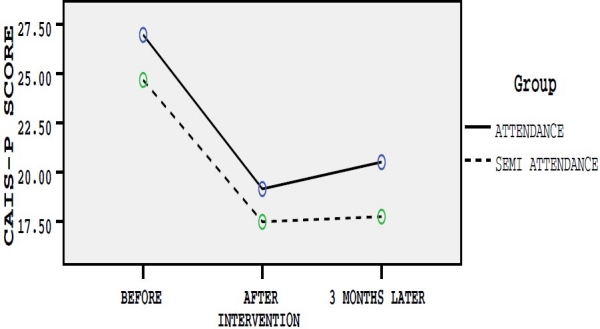
Mean scores of CAIS-P questionnaire in three different times in group A and B

Before interventions, scores of four anxiety measurement tools were considered as modifying variables and the scores for differences of changes just after and three months after intervention were considered as dependent variables and therapeutic effect of semi-attendance and attendance group therapies on dependent variables were measured. Age and education level were controlled in data analysis. The assumptions of the fitting model of multivariate analysis of covariance were checked. Box test of equality of covariance matrices and Leven’s test of equality of variances of the four dependent variables were equal across the groups (p > 0.05). Mean scores of each questionnaire based on studied groups in three different times have been presented in figures 1–4.

The results show that these differences of changes just after and three month after intervention did not differ significantly between the two groups (p > 0.05).

In overall results show that all four anxiety measurement tools had a significant difference before, just after and three months after intervention (p < 0.001) using ANCOVA Repeated Measure of analysis (details not shown). Post Hoc analysis revealed that the difference was significant between the first and second time and the first and the third time (p < 0.001) but not between the second and the third time (p = 0.771).

## Discussion

There was no statistically significant difference between the groups. In semi-attendance group CBT patients, families and therapists spent less time, the adolescents played a more active role in treatment and control of their signs, so it maybe more attractive for them and their parents. Both groups had the same levels of drop out and compliance.

CBT has been proven to be effective for treating anxiety disorders in children and adolescents.^24^,^25^ Kendall et al conducted two randomized-controlled trials demonstrating the efficacy of a 16-week individual CBT for children with anxiety disorders.^25^,^26^ Treatment gains were maintained at 1,3^27^ and 7 years post treatment.^13^ Group CBT has been shown to be effective for the treatment of anxiety in children and adolescents.^28^,^29^

Nowadays, there are studies being carried out on distance methods through computer or internet programs with optimistic results. Spence et al (2006) studied 72 children and adolescents aged from 7-14 years with anxiety disorders. Attendance group CBT and semi-attendance group CBT through internet had both significantly showed recovery signs compared to waiting list and had permanent therapeutic effect for 12 months. Semi-attendance group CBT was accepted for the families with the lowest sample drop outs.^16^

March et al (2009) surveyed 73 children aged from 7-12 years with anxiety disorders and their parents with distance group CBT through internet and then compared them with waiting list. There was a decrease in signs and an increase in function in six months follow up compared to control and 75% of the subjects became completely symptom free.^8^ In Iran, semi-attendance group CBT through media was designed instead of internet-based CBT which was effective in a three month follow up with low sample drop out. Frequent studies conducted in various fields in recent years to investigate the effect of distance group CBT in adults which have reported hopeful results in treatment of anxiety disorders,^30^,^31^ depression,^32^,^33^ panic disorder,^34^ post traumatic stress disorder,^35^ selective mutism,^36^ insomnia,^37^ cannabis and alcohol abuse^38^ and weight loss,^39^,^40^ showing the necessity of similar researches to be carried out among children and adolescents.

## Conclusions

In conclusion, present results demonstrate that both attendance and semi-attendance group CBT are effective in treatment of different kinds of anxiety disorders in adolescent girls, but still further studies and researches in this field are needed to be performed.

### 

#### Limitations

One of the main limitations to the study was the small sample size. Due to this limitation, the differences may not be shown significantly, so a study with larger sample size is suggested to be conducted. The other limitation in this study was preparation of very attractive CDs for adolescents. Although, Preparation of the CD for the first time seems expensive, but compared to the costs spent on patients’ commutation and the costs of therapist visits, semi-attendance group CBT seems somehow cost effective. Anyhow, it is a rough estimation and more studies focused on cost efficacy of this type of therapy are suggested to be conducted.
